# High prevalence of itraconazole resistance among *Candida parapsilosis* isolated from Iran

**DOI:** 10.18502/cmm.5.3.1746

**Published:** 2019-09

**Authors:** Fozieh Hassanmoghadam, Tahereh Shokohi, Mohammad Taghi Hedayati, Narges Aslani, Iman Haghani, Mojtaba Nabili, Ensieh Lotfali, Amirhossein Davari, Maryam Moazeni

**Affiliations:** 1 Student Research Committee, Mazandaran University of Medical Sciences, Sari, Iran; 2 Invasive Fungi Research Center, Mazandaran University of Medical Sciences, Sari, Iran; 3 Department of Medical Mycology, School of Medicine, Mazandaran University of Medical Sciences, Sari, Iran; 4 Infectious and Tropical Diseases Research Center, Tabriz University of Medical Sciences, Tabriz, Iran; 5 Faculty of Medicine, Sari Branch, Islamic Azad University, Sari, Iran; 6 Department of Medical Parasitology and Mycology, School of Medicine, Shahid Beheshti University of Medical Sciences, Tehran, Iran

**Keywords:** Azoles, Candida parapsilosis, Iranian isolates, Resistant

## Abstract

**Background and Purpose:**

Candida parapsilosis isolates usually have a low minimum inhibitory concentration (MIC) against azoles. Although Candida parapsilosis isolates usually have low MICs against azoles, recent studies candida invasive infections due to azole resistant-C. parapsilosis isolates . Regarding this, the main aim of this study was to determine the susceptibility pattern of Iranian clinical *C. parapsilosis* against available azole antifungal drugs.

**Materials and Methods:**

This study was conducted on 105 previously-identified isolates of *C. parapsilosis sensu stricto. *For the purpose of the study, the isolates were subjected to antifungal susceptibility testing against fluconazole (FLZ), itraconazole (ITZ), voriconazole (VRZ), and two new azole drugs, namely luliconazole (LUZU) and lanoconazole (LZN). The broth microdilution reference method adopted in this study was according to the Clinical & Laboratory Standards Institute M27-A3 and M27-S4 documents.

**Results:**

According to the results, 89% (n=94) of *C. parapsilosis* isolates showed a MIC of ≥ 1 µg/ml, indicating resistance against ITZ. Multi-azole resistance was observed in 3.8% of the isolates. In addition, LUZU and LZN demonstrated the highest efficacy with the MIC_50_ values of 0.5 and 1 µg/ml, respectively.

**Conclusion:**

The majority of the isolates showed high MIC values against ITZ. This may have been associated with the long-term ITZ prophylaxis/therapy in patients infected with candidiasis. Hence, the adoption of an appropriate antifungal agent is a crucial step for starting the treatment.

## Introduction

Incidence of invasive fungal infections due to non*-albicans Candida *species has increased, especially in immunocompromised or hospitalized patients with serious underlying diseases. The most common *Candida *species isolated from blood samples are *C. albicans *(42.1%), *C. glabrata* (26.7%), and *C. parapsilosis*, respectively [1-3]. An epidemiological study on 3,648 patients in North America showed that the proportion of candidemia caused by non*-albicans Candida* species (57.9%) was higher than that caused by *C. albicans *(42.1%). Similarly, *C. parapsilosis *has been recognized as the third common cause (34.4%) of candidemia in Iran [4]. 

*Candia parapsilosis* is a normal human commensal agent that can also live freely in environmental niches and transmit horizontally via the hands of healthcare workers and medical devices [5]. In neonates*, C. parapsilosis* is recognized as the most frequent non*-albicans Candida* species that causes invasive candidiasis [6, 7]. Among antifungal drugs, azoles and amphotericin B have been used as the main choice for the treatment of invasive candidiasis. However, new antifungal agents, such as echinocandins, have been applied as an alternative therapy in neonates [8, 9]. *Candida parapsilosis *isolates are usually reported to be susceptible to azoles. Nonetheless, the results of a recent study were indicative of the incidence of invasive *Candida* infections as a result of azoles-resistant *C. parapsilosis* isolates [10, 11]. 

Itraconazole (ITZ), a triazole antifungal agent, is a water-soluble orally active compound with a wide spectrum of antifungal activities. Recently, this agent has been used for the prophylaxis of opportunistic fungal infections, especially in patients at the risk of candidiasis, such as patients with chronic recurrent vaginal candidiasis, chronic mucocutaneous candidiasis, and AIDS, as well as those receiving immunosuppressant drugs [12]. New representatives of this class of antifungal agents (e.g., voriconazole [VRZ], posaconazole [POS], luliconazole [LUZU], and lanoconazole [LZN]) are extensively active against *Candida *species [5, 13, 14]. 

In the present study, a large number of *C. parapsilosis sensu stricto* isolates were subjected to antifungal susceptibility testing against several azole antifungals, such as fluconazole (FLZ), ITZ, VRZ, LZN, and LUZU. The aim of this study was to evaluate the susceptibility pattern of a large number of *C. parapsilosis* isolates against a comprehensive collection of available azoles. This study also involved the examination of the susceptibility pattern of *C. parapsilosis* isolates against two new antifungals, namely LUZU and LZN. 

## Materials and Methods

***Strains and Antifungal agents***

**Figure 1:**
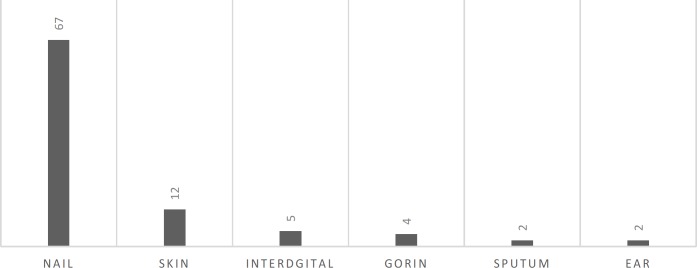
Major origins of itraconazole-resistant isolates

This study was conducted on a total of 105 *C. parapsilosis sensu stricto* strains. These species had been isolated from the different body parts of the patients infected with various clinical forms of candidiasis during 2014-2017 (Figure 1). These parts included the nails (74), hands (6), skin (2), vagina (2), urine (1), interdigital space (6), sputum (2), ear (2), and other cutaneous parts [10]. All the studied isolates were *C. parapsilosis sensu stricto* which had been previously screened by polymerase chain reaction (PCR) amplification of the secondary alcohol dehydrogenase-encoding gene (*SADH*), followed by digestion with the restriction enzyme BanI. In the mentioned investigation, *C. parapsilosis* ATCC 22019, *C. orthopsilosis* ATCC 96139, and *C. metapsilosis *ATCC 96144 were used as controls. 

Stock cultures in this study were maintained in the reference culture collection of the Invasive Fungi Research Center (IFRC, Sari, Iran). They were cultured on the 2% malt extract agar (MEA, Difco, USA) and incubated at 24°C for 2 days. The antifungal drugs (i.e., FLZ, ITZ, VRZ, LCZ, and LUZU) were in form of standard powders by their manufactures (Pfizer, Central Research, Sandwich, Kent, and the UK, respectively). The FLZ and ITZ/VRZ were resolved in sterile distilled water and dimethyl sulfide, respectively. Stoke solution of each drug was stored at -80°C.

***Antifungal susceptibility testing***

Antifungal susceptibility testing was performed according to the guidelines of the Clinical and Laboratory Standards (CLSI), M27-A3 and M27-S4 (4^th^ edition) [15]. The antifungal agents were diluted in a standard RPMI 1640 medium (Sigma Chemical Co.), and then buffered to pH 7.0 with 0.165 3-(N-Morpholino) propanesulfonic acid (MOPS, sigma chemical Co.) with l-glutamine without bicarbonate to yield two times their concentration. Subsequently, they were distributed into 96-well microdilution trays (Nunc, UK) with a final concentration of 0.016-16 µg/ml for ITZ, VRZ, LCZ, and LUZU. Regarding FLZ, this concentration was considered as 0.063-64µg/ml. 

Conidial suspensions were prepared from the isolates grown for 24 h. They were then suspended in a sterile saline solution and adjusted by spectrophotometric measurements at 530 nm wavelengths to a percent transmittance range of 75-77. A working suspension was made by a 1:10 dilution, followed by a 1:100 dilution of the stock suspension with RPMI 1640 medium, which resulted in 2.5-5×10^3^ CFU/ml. A 100-μl volume of yeast inoculum and an equal volume of antifungal agents were added to each well. Drug-free and yeast-free wells were included as positive and negative controls, respectively. The MICs were reported as the lowest drug concentration that inhibits 50% of the growth, compared to positive controls. 

The microdilution plates were incubated at 35°C and examined visually after 24 h. *Candida krusei *(ATCC6258) and *C. parapsilosis *(ATCC 22019) were used as quality controls. Based on the interpretative guidelines, the MIC values of ≤ 2, ≤ 4, and ≥ 8µg/ml were the breakpoints for: susceptible, susceptible dose-dependent (SDD), and resistant FLZ, respectively. Regarding ITZ and VRZS, these values were considered as ≤ 0.12, 0.25-0.5, and ≥ 1 µg/ml, for the aforementioned features, respectively. However, no breakpoints have been reported for LUZU and LNZ yet.

## Results and discussion

Different origins of isolates are indicated in Figure 1. Table 1 summarizes the obtained results for antifungal susceptibility testing of all 105 *C. parapsilosis* isolates. The majority of the isolates showed high MIC values against ITZ. As the results indicated, 89% (n=94) of *C. parapsilosis* isolates showed a MIC of ≥ 1 µg/ml that were ITZ-resistant according to the CLSI guideline. Sixty-seven *C. parapsilosis* isolates that were resistant to ITZ were isolated from the nails obtained from Esfahan (n=45), Mazandaran (n=25), and Tehran (n=24). The most active antifungal agent against *C. parapsilosis* isolates was VRZ, followed by LULZ, LCZ, and FLZ.

The ITZ is mainly used for the treatment of mucosal and nosocomial infections of children [16]. Clinical studies have shown that ITZ is equally efficient for the treatment of vaginal and oropharyngeal candidiasis as well [17, 18]. The growing prevalence of *C. parapsilosis* isolated from blood in neonates is associated with different environmental sources [19, 20]. Presently, the azole class of antifungals has significant advantages rendering a broad spectrum of antifungal activity and fewer side effects [21]. The widespread use of FLZ for prophylaxis, as well as empirical therapy, has been interpreted as the cause of a shift in the epidemiology of *Candida* infections. This has led to the use of other azoles for the therapy of systemic *Candida* infections [22]. 

Recent studies have shown that ITZ could be a useful alternative for FLZ resistance *Candida *species [22]. Most of the clinical *C. parapsilosis *isolates are susceptible to azoles; however, some studies have reported an increase in the incidence of invasive infections due to azole-resistance isolates [23, 24].

In this study, reduced susceptibility to ITZ was notable. Previous studies indicated that ITZ is highly active against *C. parapsilosis*. In this regard, in a study, only 3 out of 120* C. parapsilosis *isolates showed a MIC value of ≥ 1µg/ml (2.5%) against ITZ [25]. In an investigation performed on 124 medical centers worldwide, Pfaller et al. found no evidence of increasing azole resistance among C. parapsilosis isolates [26]. However, high MICs against ITZ (62%) were reported for Iranian *Candida* species isolated from the vagina [27]. 

In the present study, out of 105 *C. parapsilosis* isolates, 94 (89.5%) strains showed a MIC of ≥ 1µg/ml against ITZ. These results clearly indicate the high prevalence of ITZ resistance in Iranian *C. parapsilosis* isolates. The major origin of resistant isolates was the nail which could have been caused by the non-penetration of drugs into the nail plate [17]. It is also possible that the nail plate provides a better environment for higher biofilm formation rate. On the other hand, in recent years, ITZ has been used as an alternative drug against FLZ-resistant *Candida* isolates. Hence, long-term prophylaxis in high-risk patients can be caused by reduced ITZ susceptibility [28]. 

In conclusion, the high rate of resistance against ITZ, which is extensively used in Iran, seems to be an issue. In addition to echinocandins, awareness of the fact that *C. parapsilosis* is likely to have high MICs against ITZ seems to be a considerable issue to be addressed. It is suggested that the susceptibility pattern for isolates collected from deep candidiasis be evaluated in future studies. Moreover, further research needs to be carried out on the mechanisms of resistance.

**Table 1:** In vitro antifungal susceptibility profile of 105 clinical *Candida parapsilosis *isolates from Iran against eight antifungal agents

**Antifungal agents**	**MIC (µg/ml)**													**MIC range**	**MIC50**	**MIC90**	**GM**	**Mode**
	**≥64**	**32**	**16**	**8**	**4**	**2**	**1**	**0.5**	**0.25**	**0.125**	**0.063**	**0.031**	**0.016**					
FLZ	3	-	-	1	1	19	35	32	12	2	-	-	-	0.125-64	1	2	0.6546	1
ITZ	1	-	-	7	31	35	20	3	5	3	-	-	-	0.125-64	2	4	2.4642	2
VRZ	3	-	-	-	-	-	1	1	1	2	2	18	77	0.016-64	0.016	0.031	0.01853	0.016
LZN	-	-	-	-	8	5	13	30	22	19	6	2	-	0.063-4	0.5	2	0.35355	0.5
LUZU	-	-	-	-	20	9	26	18	12	12	8	-	-	0.063-4	1	4	1.04779	1

MIC range, geometric mean, MIC50, and MIC90 values are expressed in µg/ml

Numbers in boldfaces indicate the high MIC values

GM geometric mean MIC, MIC50 concentration at which 50 % of the isolates were inhibited, MIC90 concentration at which 90 % of the isolates were inhibit
